# Comparison of regional fat measurements by dual-energy X-ray absorptiometry and conventional anthropometry and their association with markers of diabetes and cardiovascular disease risk

**DOI:** 10.1038/ijo.2017.289

**Published:** 2018-02-06

**Authors:** S K Vasan, C Osmond, D Canoy, C Christodoulides, M J Neville, C Di Gravio, C H D Fall, F Karpe

**Affiliations:** 1 Oxford Centre for Diabetes, Endocrinology and Metabolism, University of Oxford, Churchill Hospital, Oxford, UK; 2 MRC Life-course Epidemiology Unit, University of Southampton, Southampton, UK; 3 Nuffield Department of Population Health, University of Oxford, Oxford, UK; 4 NIHR Oxford Biomedical Centre, Oxford University Hospital Trust and University of Oxford, Oxford, UK

## Abstract

**Background/Objectives:**

Fat distribution is a strong and independent predictor of type 2 diabetes (T2D) and cardiovascular disease (CVD) and is usually determined using conventional anthropometry in epidemiological studies. Dual-energy X-ray absorptiometry (DXA) can measure total and regional adiposity more accurately. Nonetheless, whether DXA provides more precise estimates of cardiovascular risk in relation to total and regional adiposity is not known. We determined the strength of the associations between DXA- and conventional anthropometry determined fat distribution and T2D and CVD risk markers.

**Subjects/Methods:**

Waist (WC) and hip circumference (HC) and DXA was used to measure total and regional adiposity in 4950 (2119 men) participants aged 29–55 years from the Oxford Biobank without pre-existing T2D or CVD. Cross-sectional associations were compared between WC and HC vs. DXA-determined regional adiposity (all *z*-score normalised) with impaired fasting glucose, hypertriglyceridemia, hypertension and insulin resistance (IR).

**Results:**

Following adjustment for total adiposity, upper body adiposity measurements showed consistently increased risk of T2D and CVD risk markers except for abdominal subcutaneous fat in both sexes, and arm fat in men, which showed protective associations. Among upper adiposity depots, visceral fat mass showed stronger odds ratios (OR) ranging from 1.69 to 3.64 compared with WC 1.07–1.83. Among lower adiposity depots, HC showed modest protection for IR in both sexes (men: OR 0.80 (95% confidence interval 0.67, 0.96); women: 0.69 (0.56, 0.86)), whereas gynoid fat and in particular leg fat showed consistent and strong protective effects for all outcomes in both men and women. The differential effect of body fat distribution on CVD and T2D were more pronounced at higher levels of total adiposity.

**Conclusions:**

Compared with DXA, conventional anthropometry underestimates the associations of regional adiposity with T2D and CVD risk markers. After correcting for overall adiposity, greater subcutaneous fat mass in particular in the lower body is protective relative to greater android or visceral adipose tissue mass.

## Introduction

Body fat distribution is associated with type 2 diabetes (T2D)^1, 2^ and cardiovascular disease (CVD)^3, 4^ independent of overall fatness. Upper body (android and visceral fat) and lower body fat depots (gynoid and leg fat) show directionally opposite associations with T2D and CVD risks.^5, 6, 7, 8, 9^ Data demonstrating the differential impact of regional fat depots on metabolic and CVD risk has been reported using either conventional anthropometric measurements^3, 5, 6, 7, 9^ or imaging methods; namely computed tomography (CT), magnetic resonance imaging (MRI) and dual-energy X-ray absorptiometry (DXA),^10, 11, 12, 13, 14, 15^ but direct comparisons of magnitude of risk between conventional anthropometry and imaging platforms have not been systematically evaluated. Circumference measurements such as waist (WC) and hip circumferences (HC) are subject to significant measurement variability and reproducibility, and this highlights the importance of quantifying body fat distribution more accurately using imaging techniques as regional adiposity has been shown to causally relate to CVD risk through multiple intermediate phenotypes.^16^

Observational studies have shown that WC and waist–hip ratio correlate strongly with total fat mass, visceral adipose tissue (VAT) and abdominal subcutaneous adipose tissue (aSAT) area quantified using CT/MRI.^7, 13, 17^ However, there is virtually no information on the relationship between HC and lower body fat depots measured using imaging methods. This is important because variation in HC includes variation in pelvic bone and gluteal muscle as well as gluteal fat, which may reduce the strength of its association with T2D and CVD risk. Higher cost, radiation exposure, variability in image analysis and logistics of using heavy equipment limit the use of CT/MRI in population studies. These problems are conveniently overcome by DXA scanning that provides accurate volumetric assessments of both total and regional adiposity.^18^ Furthermore, recent advances in DXA technology have enabled accurate quantification of VAT,^19^ thus allowing to partition android fat into VAT and aSAT, which was previously only possible using CT/MRI.

Using data from a large population-based cohort of apparently healthy British men and women, we hypothesised that precisely quantified regional adiposity measurements would display the disparate associations of different regional fat depots with T2D and CVD risk more robustly than traditional anthropometry. To achieve this, we compared the magnitude of disease risk using standardised *z*-scores of regional fat measurements quantified using anthropometry and DXA thus enabling direct comparisons of risk estimates.

## Subjects and Methods

### Study participants

This cross-sectional study included 4950 participants (2119 men and 2831 women) aged between 29 and 55 years from the Oxford Biobank (OBB; http://www.oxfordbiobank.org.uk/). Details of the OBB and study recruitment are described elsewhere.^20, 21^ In brief, men and women without any known chronic disease residing in Oxfordshire, UK, and selected randomly from the UK National Health Service population register participated in a clinic assessment, which included collection of lifestyle information (smoking, alcohol use, physical activity) using questionnaires, biochemical testing, anthropometric measurements and body composition analysis using DXA. At the clinic visit, trained healthcare professionals measured the participants’ standing height, weight, WC and HC of each participant using standardised equipment and protocols. WC and HC measurements were recorded by research nurses trained to a standard operating procedure based on the WHO Steps manual (http://www.who.int/chp/steps/manual/en/) to ensure reproducible measurements and recognition of correct anatomical landmarks. The between-observer coefficient of variation for HC was 1.1% (95% confidence interval (CI) was 0.9–1.4%) and 1.8% (95% CI 1.4–2.1%) for WC. Body mass index (BMI) was calculated as weight/height^2^. Fasting plasma glucose, triglycerides, total plasma cholesterol and high-density lipoprotein-cholesterol were measured enzymatically using commercially available kits on an ILab 650 clinical analyzer (Instrumentation Laboratory, UK). Low-density lipoprotein-cholesterol was calculated using the Friedewald formula. Plasma insulin concentrations were measured by radioimmunoassay (Millipore, Watford, UK Ltd.). Insulin resistance was calculated using the homoeostatic model assessment insulin resistance equation (HOMA-IR).^22^ Blood pressure was recorded using an automated pulse-detecting sphygmomanometer (Omron M3). Four readings were recorded, and the mean of the last three measurements was used in the analysis. All study-related procedures were carried out at the clinical research unit at the Oxford Centre for Diabetes, Endocrinology and Metabolism, in Oxford, UK. The study was approved by the Oxford Ethics Committee, and all participants gave consent to participate in the study.

### Body composition by DXA

Body composition was assessed using GE Lunar iDXA machine, which has excellent precision for body composition estimates and good concordance with CT for VAT mass.^23, 24^ The regions of interest were automatically defined by Encore software (version 14.0; GE Healthcare, Bucks, UK) and described in detail elsewhere.^25^ All DXA measurements were recorded on a single GE Lunar DXA scanner throughout the study operated by trained staff according to standard operating procedure. The machine’s calibration was performed every morning of the day with scanning using a GE Lunar calibration phantom. Scans were not performed unless calibration passed the quality control. Regions of interest were (i) Android fat—the area of the abdomen from a line joining the two superior iliac crests and extended cranially for 20% of the distance between to the base of the skull; (ii) Gynoid fat—the portion of the legs from the greater femoral trochanter, extending caudally to mid-thigh; and (iii) Leg fat—the area of the entire leg (partially overlaps with gynoid fat). Estimated VAT mass, was not a direct measurement and calculated using a specific algorithm as described elsewhere and has been show to strongly correlate with CT-measured VAT (*r*^2^=0.957).^19^ The validation population described by Kaul *et al.* s ethnically similar to the OBB participants and within the age and BMI range. In brief, the algorithm uses X-ray attenuation values of total abdominal thickness and the subcutaneous fat width along the lateral aspect of the abdomen along with empirically derived geometric constants to estimate the quantity of aSAT in the android region. VAT is then computed by subtracting aSAT from fat in the total android region. The aSAT mass was calculated as the difference between android fat mass and VAT. Fat mass index (FMI) was calculated as total fat mass (kg) divided by height (m) squared (kg m^−2^).

### Outcome definitions

The main outcomes were defined as follows: (i) impaired fasting glucose: fasting plasma glucose⩾5.6 mmol; (ii) hypertension: systolic blood pressure⩾140 mmHg or diastolic blood pressure⩾90 mmHg, mean arterial blood pressure was calculated as 1/3 (systolic blood pressure–diastolic blood pressure)+diastolic blood pressure; (iii) hypertriglyceridemia: plasma triglycerides⩾1.5 mmol l^−1^ and (iv) insulin resistance (IR) was defined as >75th percentile (Male⩾4.19; Female⩾3.42) HOMA-IR for the study population.

### Statistical methods

**Figure 1:**
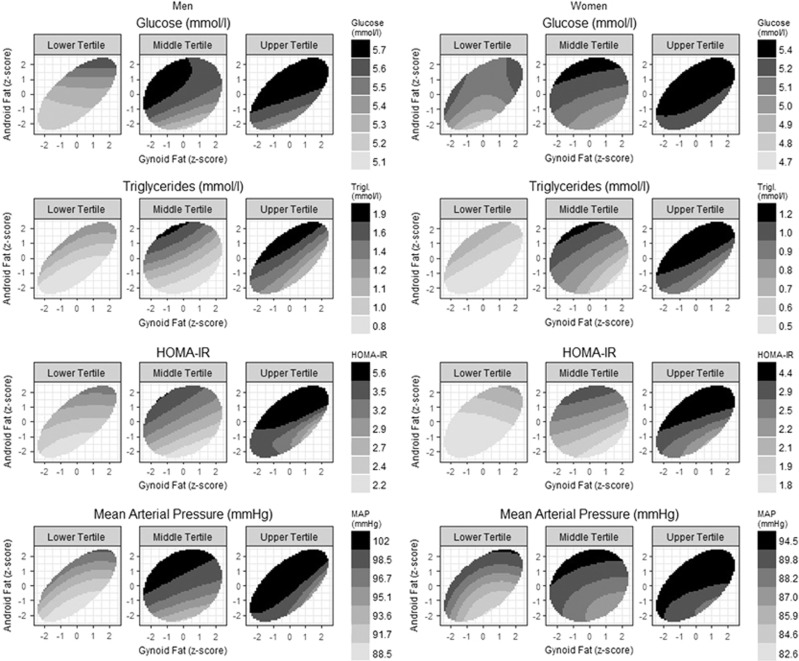
Relationship between android and gynoid fat mass across fat mass index tertiles with mean arterial pressure and metabolic traits. Tertiles of fat mass index for men; lower<5.86 kg m^−^^2^; middle 5.86–8.02 kg m^−2^ and upper tertile>8.02 kg m^−2^. Tertiles of fat mass index for women; lower<7.02 kg m^−2^; middle 7.02–9.80 kg m^−2^ and upper tertile>9.80 kg m^−^^2^.

The characteristics of the study participants are presented as median (interquartile range; IQR) for continuous variables and *n* (percentage, %) for categorical variables. Pairwise associations between the anthropometric and DXA-derived regional fat measurements depots were analysed using Pearson correlations. Partial correlations adjusted for BMI and FMI are also presented to understand how total adiposity influences the relationship between anthropometry and DXA-derived adiposity depots. By using height as a denominator in both BMI and FMI, they potentially eliminate the presumed relationship between fat mass and stature.^26^ Multivariate logistic regression analysis was used to investigate the relationship of regional adiposity measurements with dichotomous T2D and CVD risk factor outcomes. As the anthropometric and DXA-derived measures were recorded in different units, they were converted into age- and sex-specific *z*-scores using Fisher Yates transformation.^27^ This approach allows direct comparison of risk magnitude per 1 standard deviation (s.d.) change difference in the adiposity measurement. We present the odds ratio (OR) for each outcome per s.d. change in the adiposity measure. Another advantage of using *z*-scores is that it allows direct comparison of the magnitude of association across different adipose depot measurements and outcomes. We present two models: (1) Model 1: adjusted for age, physical activity, smoking and alcohol intake (2) Model 2: adjusted additionally for either BMI (when analysing WC and HC) or FMI (when analysing DXA depots). Owing to significant interaction with sex in all models (*P*<0.05), we analysed the data separately for men and women. All models were tested with variation inflation factor statistics and none of the factors were affected by multicollinearity. STATA version 11.2 (College Station, TX, USA) software was used to analyse the data. Finally, we derived contour plots, using R software (V.3.3.1), to illustrate the separate and combined effects of android, gynoid and total fat on risk factors (Figure 1). We used the Mahalanobis distance to identify an ellipse containing 95% of the data for each plot, to ensure that contour lines were based only on real data.

## Results

Characteristics of the study participants are summarised in Table 1. Men and women had a median (interquartile range) age of 43 (37, 46) and 42 (37, 46) years, respectively. About 90% of subjects were non-smokers. As expected men had significantly greater WC and greater android fat mass than women, whereas women had significantly greater gynoid and leg fat mass than men. There was no significant difference in HC between sexes (*P* =0.16). Higher BMI was associated with higher WC, HC and DXA-quantified total fat mass in both sexes (Supplementary Table 1). Comparing subjects in the highest and lowest quintiles of BMI, total fat mass and all fat depots except VAT were approximately twofold higher, whereas VAT mass was sixfold higher in men and 10-fold higher in women. These large obesity-driven variations in DXA-determined VAT depot size were clearly not captured by changes in WC.

The correlations between WC, HC, height, BMI, FMI and lean body mass (LBM) are shown in Table 2. Overall, the DXA-measured adiposity measurements were directionally positive, strongly correlated and generally in the same order for WC, HC and BMI. Height was weakly related to regional and total fat masses. We found particularly strong correlations between BMI-unadjusted WC and android fat (*r=*0.91 in men, *r*=0.87 in women), and between BMI-unadjusted HC and gynoid fat (*r=*0.81 in men, *r=*0.89 in women). However, BMI-unadjusted WC and HC also correlated equally strongly with total fat mass (WC: *r=*0.90 in men, *r=*0.85 in women; HC: *r=*0.80 in men, *r=*0.88 in women), which made it difficult to establish if WC and HC reflect the size of their respective distinct regional fat depots when total adiposity is not accounted for. Following adjustments for age and either BMI or FMI, the correlations between anthropometry and DXA-measurements showed an overall attenuation. Nevertheless, the adjusted partial correlations remained strong for WC and android fat in both sexes (BMI adjusted: *r=*0.62 in men, *r=*0.47 in women; FMI adjusted: *r=*0.51 in men, *r=*0.44 in women) and HC and gynoid fat in both sexes (BMI adjusted: *r=*0.49 in men, *r=*0.61 in women; FMI adjusted: *r=*0.54 in men, *r=*0.56 in women). LBM showed an overall weaker correlation with DXA-fat depots.

Table 3 (Model 1) shows the associations between regional fat depot size and T2D and CVD risk factors. In the BMI and FMI unadjusted analyses, we found uniformly higher ORs for DXA-based measurement compared with WC, with highest OR observed with VAT.

Following adjustment for BMI and other covariates (Model 2), the ORs for WC associations with cardiometabolic risk factors were attenuated in both sexes, and became nonsignficant for impaired fasting glucose (OR 1.07; 95% CI 0.86, 1.34) and hypertension [(1.16; (0.88, 1.53)] in men. The ORs were 1.5- to threefold stronger for DXA-quantified depots compared with anthropometry. FMI adjusted android fat mass was associated with an overall increased ORs for T2D and CVD risk factors in both men and women ranging from 1.93 to 5.01. The strongest risk association observed for 1 s.d. (~1 kg in men and women) increase in android fat mass was with hypertriglyceridemia (men: 5.01 (3.25, 7.69); women: 4.15 (2.52, 6.82)) and IR (men: 3.89 (2.51, 6.02); women: 3.43 (2.27, 5.18)). When the risk associated with android fat components vs VAT mass and aSAT were compared, these two fat depots showed distinctly different associations with T2D and CVD risk factors. Whereas VAT displayed an overall increased risk (OR ranging from 1.69 to 3.64), aSAT showed ORs below one indicating a protective role of subcutaneous tissue (OR ranging from 0.55 to 0.73), except for IR in women. Arm fat was also associated with protective ORs for all outcomes (OR ranging from 0.48 to 0.73), but significant only in men.

Unadjusted measures of lower body adiposity (HC, gynoid fat and leg fat) were positively associated with an adverse T2D and CVD risk factor profile in both men and women (Table 3, Model 1). Following adjustment with either BMI or FMI, the associations between lower body adiposity measurements and CVD/metabolic risk became directionally opposite and significantly protective for most of the outcomes for gynoid and leg fat in both sexes. The strongest risk reductions were observed with hypertriglyceridemia. A 1 s.d. (equivalent to ~1.5 kg) increase in leg or gynoid fat mass was associated with substantially lower risk of hypertriglyceridemia: (leg fat: OR (95% CI), men: 0.32 (0.25, 0.41); women: 0.30 (0.23, 0.40) and gynoid fat, men: 0.44 (0.34, 0.58); women: 0.25 (0.18, 0.35)). In contrast BMI-adjusted HC showed a significantly protective effect only for IR in both sexes (OR (95% CI) men: 0.80 (0.67, 0.96); women: 0.69 (0.56, 0.86)) and with hypertriglyceridemia in women (OR 0.48; 95% CI 0.38, 0.63). Although WC and HC associate strongly with the fat masses in the respective regions, these measures also include bone structure and lean mass in the region. We therefore tested if there was an association between LBM and the risk markers. Overall, LBM showed a modestly increased OR for all the outcomes. However, when adjusted for FMI, the associations were no longer statistically significant implying that the risk is probably driven by a collinearity between LBM and total body fatness. Also, LBM, as a component of what is measured by WC and HC, is unlikely to affect the association between anthropometric circumference measurements and the respective diabetes and cardiovascular risk and the effects are primarily due to fat components measured by WC and HC.

In order to simultaneously analyse the interactions between total fat, android and gynoid fat in relation to continuous measures of risk factors, we constructed contour plots of risk factors according to android and gynoid fat, stratified by tertiles of FMI (Figure 1). In both men and women, an increase in gynoid fat mass at any level of android fat, was generally associated with lower fasting glucose, triglycerides, HOMA-IR and mean arterial blood pressure. This was most evident in the highest FMI tertile as indicated by more vertical contour lines. Taking triglyceride concentrations as an example, the highest triglyceride concentrations were among men and women who were in the highest tertile of FMI, and who had high android fat but low gynoid fat. In contrast, the interaction of the opposing associations between android and gynoid fat masses was less clear in the lowest tertile of FMI. At higher FMI tertiles, even a small s.d. change in either gynoid or android fat mass was associated with directionally opposite effects in the outcomes, whereas at lowest tertile, the protective effect observed with gynoid fat depot was not evident although the risk with android fat was consistent across all tertiles.

## Discussion

This study was designed to examine cross-sectional associations between conventional anthropometric vs. DXA-quantified regional fat masses and a range of range of T2D and CVD risk factors. We demonstrate that (i) WC and HC are imprecise surrogates of regional adiposity as they also reflect total fat (ii) DXA-measured fat masses are strongly associated with T2D and CVD risk factors and bring out the dichotomy between upper and lower body adiposity in relation to cardiac and metabolic risk more robustly than conventional anthropometry (iii) Subcutaneous fat depots are associated with a favourable cardiometabolic profile with modest associations observed with arm fat and aSAT (iv) the opposing associations of upper and lower fat depots with risk factors are more evident at higher levels of total adiposity.

In a systematic approach to understanding the relationship between anthropometry and DXA-assessed regional fat masses and T2D and CVD risk factors, we first sought to understand how the measurements of regional adiposity assessed by two different instruments relate to each other. In unadjusted analysis, both WC and HC strongly correlated with their corresponding regional fat depots, that is, android and gynoid fat mass respectively. WC displayed equally strong correlations with aSAT, VAT, android fat and total fat mass which makes it difficult to disentangle the specific regional fat depot that it actually reflects. However, these associations were markedly and uniformly attenuated following adjustment for total adiposity. Furthermore, our results suggest that the risk associations of WC with CVD and T2D traits is not contingent upon the context of VAT alone, but rather represent the heterogeneity in the adipose tissues within the abdominal region. In this respect, emerging evidence supported by our findings shows that aSAT and VAT are morphologically and functionally different.^28, 29^ Adding further complexity aSAT can be divided into two distinct layers separated by Scarpa’s fascia, that is, superficial and deep aSAT, which have functional differences and also show distinct associations in relation to metabolic risk and hypertension.^30^ Similarly, HC demonstrates strong correlation not only with gynoid and leg fat, but also with total fat again reflecting that these anthropometric circumference measurements are surrogates of total adiposity and not exclusively regional fat.

Previous studies have shown that WC and HC have opposite associations with CVD and metabolic risk factors,^3, 6, 7, 8, 9, 31^ but these were evident only when both WC and HC were included in the same model. Similarly, regional fat depots measured using imaging techniques have also been previously shown to relate paradoxically with cardiometabolic risks.^10, 14, 32^ However, to our knowledge there are no studies that have attempted to compare the disease risk magnitudes of regional adiposity measured by imaging techniques with WC and HC. In that respect, ours is the first study to include comprehensive DXA measures of regional adiposity, including aSAT and VAT, to make a comparison between anthropometric and DXA measures in relation to cardiometabolic risk factors and our results highlight that more precise DXA estimations of regional adiposity bring out the cardiometabolic risk more robustly compared with conventional anthropometry.

The association of VAT mass with a more adverse T2D and CVD risk profile is consistent with previous reports based on CT or MRI acquired VAT data.^29, 33, 34^ We provide the first report of DXA-measured VAT-associated risk on disease risk factor outcomes. VAT and android fat remained robustly associated with CVD and T2D risk factors after accounting for FMI. However, the component of android fat mass that is not VAT (aSAT) showed a ‘paradoxical’, albeit moderate, protection against T2D and CVD risk factors suggesting that the relative partitioning of the central fat depot into subcutaneous and visceral component are important determinants of cardiometabolic health. This finding is consistent with a recent study by McLaughlin *et al.,*^35^ which showed that CT-measured abdominal SAT was inversely related to IR and with the results of the Framingham Heart study where individuals in highest tertiles of aSAT had significantly lower risk of metabolic syndrome.^36^ The protective association we observed with arm fat on FMI adjustment is novel. We speculate that the arm compartment in men (who generally have less lower body fat compared with women) functions somewhat similar to the leg fat depot in women, acting as an additional reservoir for subcutaneous fat storage. Against this interpretation, a case-control study from and the Korean National Health and Nutrition examination survey, which showed that BMI-adjusted arm fat was associated with an increased risk of T2D, particularly in women.^14^ Reason for this discrepancy may be related to inclusion of older men aged >50 years and post-menopausal women who are generally prone to age-related loss in subcutaneous fat.

The protective associations of the subcutaneous and lower fat depots were apparent only after adjustment for overall adiposity (FMI) and again more consistent for DXA-quantified aSAT, gynoid and leg depots compared with HC. In other words, greater adiposity is associated with greater cardiometabolic risk, whatever its location, but at any level of total body fat, having a greater proportion of that fat in subcutaneous and lower body compartment is beneficial. The lower body DXA measurements showed stronger associations with risk factors than HC, probably mainly because it eliminates the contribution by muscle mass in the gluteofemoral and leg region, which is inherent in HC measurement. Concordant with previous reports, we demonstrated that gynoid and leg fat are associated with a more favourable metabolic profile.^10, 11, 14, 37, 38^ This beneficial relationship of having a greater proportion of fat in the lower depots may indicate a greater lower body fat reservoir and thus protection from ectopic fat deposition. The protective associations were slightly stronger in women suggesting that they have increased propensity to store fat safely or effectively in the lower body fat compartments. We recently presented a coherent view to explain the differences in metabolic properties between abdominal and gluteofemoral adipose tissue concluding that the specific function of the lower body depots impacts whole-body metabolism.^39^

The contour plots illustrate the differential association of upper and lower body adiposity with continuous metabolic traits, and suggest that these are more important at high overall levels of adiposity. At lower levels of adiposity, body fat distribution does not appear to impact strongly on CVD and T2D risk factors

We recognise some of the strengths and limitations of our study. The OBB study participants represent a homogenous, relatively healthy population of white Caucasians who are representative of the UK population. They have a wide range of age (29–55 years), BMI (16–49 kg m^−2^) and central adiposity (WC range 59–143 cm) thus enabling us to undertake robust comparisons across a wide range of body phenotypes. However, we acknowledge that our results cannot be immediately extended to other populations with different body fat patterning. The study is cross-sectional in design and therefore does not allow inference of causal relationships. Trained staff measured WC and HC and the intra-observers variation for these measurements were low. Estimated VAT was not calculated from predictive equations, but based on an automated algorithm from the DXA manufacturer providing valid estimates of this particular fat depot.

In conclusion, our study provides evidence that conventional anthropometry offers information regarding both fat distribution and total adiposity, but provides weaker predictions of T2D and CVD risk factors than DXA measures. After accounting for overall adiposity, VAT is associated with higher risk factors, whereas gynoid, leg, arm and aSAT is associated with lower risk factors. The differential associations support the ‘adipose tissue expandability’ hypothesis, that sufficient storage of excessive fat in subcutaneous compartments particularly in lower body depots is cardio-metabolically protective and in its absence, android and VAT is detrimental.^40^

## Figures and Tables

**Table 1:** Characteristics of the Oxford Biobank participants

	*Men (*n=*2119)*	*Women (*n=*2831)*
Age (years)	43 (37, 46)	42 (37, 46)
Non-smokers, % (*n*)	89.1 (1185)	91.7 (2593)
		
*Alcohol consumption*,^a^*% (*n)		
None	0.9 (20)	3.2 (89)
Moderate consumption	86.6 (1835)	88.5 (2505)
Heavy consumption	12.5 (264)	8.3 (236)
		
*Physical activity, % (*n)		
Sedentary	4.2 (89)	4.3 (121)
Moderately active	55.8 (1181)	70.1 (1983)
Active	40.0 (845)	25.6 (724)
		
*Anthropometry*		
Height (cm)	179 (175, 183)	165 (161, 170)
Weight (kg)	83.9 (76.1, 93.5)	66.7 (60.1, 75.8)
Body mass index (kg m^−2^)	26.1 (23.8, 28.8)	24.2 (21.9, 27.7)
Waist circumference (cm)	92 (85, 100)	80 (73, 89)
Hip circumference (cm)*	101 (97, 106)	100 (95, 106)
Waist–hip ratio	0.91 (0.87, 0.96)	0.80 (0.76, 0.86)
		
*DXA measurements*		
Android fat (kg)	2.1 (1.4, 2.9)	1.6 (1.1, 2.5)
Visceral fat (kg)	0.9 (0.5, 1.6)	0.3 (0.1, 0.6)
Abdominal subcutaneous fat (kg)	1.0 (0.8, 1.4)	1.3 (0.9, 1.8)
Arm fat (kg)	2.2 (1.7, 2.8)	2.6 (2.0, 3.3)
Gynoid fat (kg)	3.3 (2.6, 4.1)	4.3 (3.4, 5.4)
Leg fat (kg)	6.0 (4.8, 7.5)	8.5 (6.9, 10.7)
Total fat (kg)	22.0 (16.9, 28.1)	22.7 (17.6, 29.6)
Total lean body mass (kg)	57.7 (53.5, 62.5)	41.1 (37.9, 44.5)
		
*Metabolic traits*		
Fasting glucose (mmol l^−1^)	5.4 (5.1, 5.7)	5.0 (4.8, 5.3)
Fasting insulin (mU l^−1^)	12.3 (9.4, 16.4)	10.7 (8.2, 14.2)
HOMA-IR	2.9 (2.2, 4.0)	2.4 (1.8, 3.2)
Total cholesterol (mmol l^−1^)	5.2 (4.5, 5.9)	5.0 (4.3, 5.6)
Triglycerides (mmol l^−1^)	1.2 (0.8, 1.7)	0.8 (0.6, 1.1)
HDL cholesterol (mmol l^−1^)	1.2 (1.0, 1.4)	1.5 (1.2, 1.8)
LDL cholesterol (mmol l^−1^)	3.4 (2.8, 3.9)	3.0 (2.5, 3.5)
Systolic blood pressure (mmHg)	126 (119, 134)	115 (107, 123)
Diastolic blood pressure (mmHg)	79 (73, 85)	72 (67, 79)
Mean arterial blood pressure (mmHg)	94 (89, 101)	86 (81, 93)

Abbreviations: DXA, dual-energy X-ray absorptiometry; HDL, high-density lipoprotein-cholesterol; HOMA-IR, homoeostatic model assessment insulin resistance; LDL, low-density lipoprotein-cholesterol. Data presented as median (interquartile range) for continuous variables and percentage (frequency) for categorical variables.

a Weekly alcohol consumption was categorised as: no alcohol intake, Moderate (<14 units for women, <21 units for men); Heavy (15–35 units for women, 22–50 units for men).

**Table 2:** Correlations between total adiposity and fat distribution measured by anthropometry and dual-energy X-ray absorptiometry

	*Unadjusted correlations*						*Adjusted for age and BMI*		*Adjusted for age and FMI*	
	*Waist*	*Hip*	*Height*	*BMI*	*FMI*	*Lean body mass*	*Waist*	*Hip*	*Waist*	*Hip*
*Men*										
Total fat	0.9	0.8	0.13	0.86	0.98	0.32	0.58	0.38	0.44	0.48
Android	0.91	0.76	0.07	0.86	0.97	0.29	0.62	0.26	0.51	0.23
Visceral fat	0.85	0.65	0.02*	0.8	0.86	0.28	0.49	0.06	0.36	0.04
Abdominal SAT	0.75	0.71	0.14	0.7	0.84	0.17	0.42	0.34	0.1	0.22
Arm fat	0.83	0.76	0.12	0.81	0.92	0.31	0.39	0.31	0.1	0.26
Gynoid fat	0.82	0.81	0.18	0.79	0.92	0.29	0.44	0.49	0.1	0.54
Leg fat	0.76	0.77	0.18	0.74	0.88	0.26	0.36	0.43	−0.03	0.33
										
*Women*										
Total fat	0.85	0.88	0.06	0.91	0.98	0.27	0.35	0.47	0.32	0.51
Android	0.87	0.81	−0.01*	0.88	0.96	0.21	0.47	0.25	0.44	0.06
Visceral fat	0.76	0.63	−0.07	0.75	0.79	0.18	0.35	0.01*	0.3	−0.07
Abdominal SAT	0.84	0.82	0.01*	0.87	0.94	0.15	0.4	0.32	0.31	0.17
Arm fat	0.81	0.81	0.06	0.86	0.93	0.27	0.29	0.3	0.19	0.18
Gynoid fat	0.74	0.89	0.12	0.84	0.92	0.28	0.1	0.61	−0.08	0.56
Leg fat	0.68	0.85	0.13	0.8	0.87	0.28	0.02	0.54	−0.14	0.45

Abbreviations: BMI, body mass index; FMI, fat mass index. Pearson correlations using transformed measures (*z*-scores); anthropometric and DXA-measured fat depots. All correlations are statistically significant at *P*<0.001 except *non-significant.

**Table 3:** Measures of fat distribution and lean body mass association with impaired fasting glucose, hypertriglyceridemia, hypertension and insulin resistance in men and women

	*Z-Waist OR (95% CI)*	*Z-Android fat OR (95% CI)*	*Z-Visceral fat OR (95% CI)*	*Z- abdominal SAT OR (95% CI)*	*Z-arm fat OR (95% CI)*	*Z-Hip OR (95% CI)*	*Z-Gynoid fat OR (95% CI)*	*Z-Leg fat OR (95% CI)*	*Z-lean body mass OR (95% CI)*
*MODEL 1*									
Men (*n*=2119)									
** **Impaired fasting glucose (*n*=626)	1.62 (1.46, 1.80)	1.71 (1.53, 1.90)	1.79 (1.60, 2.01)	1.39 (1.26, 1.55)	1.41 (1.28, 1.56)	1.39 (1.26, 1.54)	1.51 (1.36, 1.68)	1.37 (1.24, 1.52)	1.18 (1.06, 1.31)
** **Hypertriglyceridemia (*n*=661)	2.48 (2.20, 2.79)	2.78 (2.45, 3.15)	3.27 (2.85, 3.76)	1.69 (1.51, 1.87)	2.10 (1.87, 2.34)	1.81 (1.63, 2.02)	1.95 (1.74, 2.17)	1.66 (1.49, 1.84)	1.32 (1.18, 1.46)
** **Hypertension (*n*=377)	1.88 (1.66, 2.14)	1.95 (1.70, 2.23)	2.17 (1.89, 2.50)	1.41 (1.24, 1.59)	1.56 (1.37, 1.76)	1.55 (1.37, 1.75)	1.51 (1.33, 1.72)	1.36 (1.20, 1.53)	1.16 (1.02, 1.31)
** **Insulin resistance (*n*=647)	2.74 (2.41, 3.10)	3.68 (3.21, 4.23)	3.16 (2.74, 3.62)	2.01 (1.80, 2.26)	2.11 (1.88, 2.36)	1.92 (1.72, 2.14)	2.21 (1.97, 2.49)	1.86 (1.67, 2.09)	1.39 (1.24, 1.54)
Women (*n*=2831)									
** **Impaired fasting glucose (*n*=285)	2.21 (1.90, 2.53)	2.33 (2.01, 2.69)	2.58 (2.21, 3.01)	1.98 (1.72, 2.28)	1.94 (1.69, 2.23)	1.89 (1.65, 2.17)	1.82 (1.58, 2.08)	1.59 (1.39, 1.82)	1.32 (1.14, 1.53)
** **Hypertriglyceridemia (*n*=281)	2.68 (2.31, 3.11)	2.91 (2.49, 3.39)	3.44 (2.92, 4.06)	2.32 (2.01, 2.68)	2.34 (2.02, 2.71)	1.91 (1.67, 2.19)	1.83 (1.59, 2.09)	1.63 (1.43, 1.86)	1.33 (1.15, 1.54)
** **Hypertension (*n*=194)	1.95 (1.66, 2.29)	1.96 (1.67, 2.32)	2.18 (1.84, 2.58)	1.71 (1.45, 2.01)	1.73 (1.47, 2.02)	1.64 (1.40, 1.92)	1.48 (1.27, 1.73)	1.33 (1.13, 1.54)	1.03 (0.86, 1.22)
** **Insulin resistance (*n*=505)	3.43 (3.00, 3.92)	3.03 (2.64, 3.46)	4.03 (3.48, 4.66)	2.99 (2.63, 3.39)	2.95 (2.60, 3.35)	2.56 (2.27, 2.89)	2.45 (2.18, 2.76)	2.06 (1.83, 2.30)	1.37 (1.22, 1.54)
									
*MODEL 2*									
** **Men (*n*=2119)									
** **Impaired fasting glucose (*n*=626)	1.07 (0.86, 1.34)	1.93 (1.30, 2.88)	1.69 (1.36, 2.11)	0.73 (0.61, 0.87)	0.56 (0.43, 0.72)	0.82 (0.69, 1.02)	0.79 (0.61, 1.03)	0.61 (0.49, 0.76)	1.07 (0.95, 1.20)
** **Hypertriglyceridemia (*n*=661)	1.83 (1.45, 2.32)	5.01 (3.25, 7.69)	3.64 (2.82, 4.70)	0.56 (0.47, 0.67)	0.73 (0.57, 0.96)	0.89 (0.75, 1.06)	0.44 (0.34, 0.58)	0.32 (0.25, 0.41)	1.10 (0.98, 1.25)
** **Hypertension (*n*=377)	1.16 (0.88, 1.53)	2.65 (1.63, 4.31)	2.49 (1.88, 3.31)	0.55 (0.45, 0.67)	0.58 (0.43, 0.79)	0.91 (0.75, 1.11)	0.44 (0.32, 0.61)	0.38 (0.29, 0.50)	0.97 (0.84, 1.13)
** **Insulin resistance (*n*=647)	1.54 (1.21, 1.98)	3.89 (2.51, 6.02)	2.36 (1.83, 3.02)	0.70 (0.57, 0.84)	0.48 (0.36, 0.63)	0.80 (0.67, 0.96)	0.57 (0.42, 0.75)	0.39 (0.31, 0.50)	1.14 (0.92, 1.29)
** **Women (*n*=2831)									
** **Impaired fasting glucose (*n*=285)	1.40 (1.06, 1.85)	2.69 (1.67, 4.34)	2.39 (1.83, 3.13)	0.59 (0.41, 0.87)	0.76 (0.55, 1.06)	0.81 (0.62, 1.05)	0.57 (0.42, 0.78)	0.50 (0.38, 0.64)	1.07 (0.91, 1.28)
** **Hypertriglyceridemia (*n*=281)	1.63 (1.23, 2.18)	4.15 (2.52, 6.82)	3.57 (2.67, 4.78)	0.63 (0.43, 0.93)	0.88 (0.63, 1.23)	0.48 (0.38, 0.63)	0.25 (0.18, 0.35)	0.30 (0.23, 0.40)	1.09 (0.91, 1.31)
** **Hypertension (*n*=194)	1.41 (1.02, 1.94)	2.38 (1.37, 4.16)	2.22 (1.63, 3.02)	0.61 (0.38, 0.93)	0.87 (0.59, 1.29)	0.84 (0.62, 1.15)	0.41 (0.28, 0.58)	0.41 (0.31, 0.55)	0.88 (0.72, 1.08)
** **Insulin resistance (*n*=505)	1.75 (1.38, 2.23)	3.43 (2.27, 5.18)	2.84 (2.24, 3.60)	0.82 (0.59, 1.13)	0.95 (0.71, 1.26)	0.69 (0.56, 0.86)	0.43 (0.33, 0.56)	0.36 (0.29, 0.45)	1.13 (0.98, 1.31)

Estimates presented as odds ratios (OR) and 95% confidence interval (CI) and represent risk of outcome with 1 s.d. increase in adiposity measurement. ^**†**^Model 1: adjusted for age, smoking, physical activity, alcohol intake. ^**‡**^Model 2: adjusted for age, smoking, physical activity, alcohol intake and BMI for waist and hip circumferences/FMI for DXA-derived depots.
