# Supplementary material for: Comparison of regional fat measurements by dual-energy X-ray absorptiometry and conventional anthropometry and their association with markers of diabetes and cardiovascular disease risk
Source: Int J Obes (Lond). 2018 Feb 6;42(4):850–7. doi: 10.1038/ijo.2017.289 (PMC5965665; doi:10.1038/ijo.2017.289)
Supplement: Supplementary Table 1 [file ijo2017289x1.docx]

**Supplementary table 1: Fat measurements according to quintiles of body mass index, and percentage difference in fat measures between the lowest and highest BMI quintiles**

|  | **Quintile 1** | | **Quintile 2** | | **Quintile 3** | | **Quintile 4** | | **Quintile 5** | | **%difference**  **Q5/Q1** |
| --- | --- | --- | --- | --- | --- | --- | --- | --- | --- | --- | --- |
| **Men (n=2,119)** |  | |  | |  | |  | |  | |  |
| **Body mass index (kg/m2)** | 21.1 | (20.3, 21.7) | 23.2 | (22.7, 23.7) | 25.2 | (24.6, 25.7) | 27.5 | (26.8, 28.3) | 31.4 | (30.1, 33.6) | 1.5 |
| Weight (kg) | 67.2 | (63, 72.4) | 74.7 | (71.1, 79.1) | 80.8 | (76.8, 84.6) | 88.4 | (84, 93.5) | 101.3 | (95, 109) | 1.5 |
| Waist circumference (cm) | 78 | (75, 82) | 84 | (80, 88) | 90 | (86, 92) | 96 | (92, 99) | 106 | (101, 112) | 1.4 |
| Hip circumference (cm) | 92 | (90, 96) | 97 | (94, 99) | 100 | (97, 103) | 103 | (100, 106) | 109 | (106, 113) | 1.2 |
| Android fat (kg) | 0.8 | (0.6, 1.1) | 1.3 | (1.0, 1.7) | 1.8 | (1.5, 2.2) | 2.5 | (2.0, 2.9) | 3.6 | (3.0, 4.2) | 4.5 |
| Visceral fat (kg) | 0.3 | (0.2, 0.4) | 0.5 | (0.3, 0.7) | 0.8 | (0.5, 1.1) | 1.2 | (0.9, 1.6) | 2.0 | (1.5, 2.5) | 6.7 |
| Abdominal subcutaneous fat (kg) | 0.6 | (0.3, 0.8) | 0.8 | (0.6, 1.0) | 1.0 | (0.8, 1.2) | 1.2 | (1.0, 1.4) | 1.6 | (1.3, 2.0) | 2.7 |
| Arm fat (kg) | 1.3 | (1.0, 1.6) | 1.7 | (1.4, 2.0) | 2.0 | (1.7, 2.3) | 2.4 | (2.1, 2.8) | 3.3 | (2.8, 3.8) | 2.5 |
| Gynoid fat (kg) | 2.1 | (1.7, 2.5) | 2.6 | (2.2, 3.1) | 3.1 | (2.6, 3.5) | 3.6 | (3.1, 4.1) | 4.7 | (4.1, 5.5) | 2.2 |
| Leg fat (kg) | 4.1 | (3.2, 4.9) | 4.8 | (4.0, 5.7) | 5.6 | (4.8, 6.5) | 6.4 | (5.5, 7.5) | 8.5 | (7.2, 10.1) | 2.1 |
| Total fat (kg) | 12.4 | (10.2, 14.7) | 16.3 | (13.3, 19.1) | 20.3 | (17.3, 22.7) | 24.6 | (21.7, 27.6) | 32.8 | (29.2, 38.1) | 2.6 |
| Total lean body mass (kg) | 50.9 | (47.3, 54.8) | 54.8 | (51.1, 58.1) | 56.6 | (53.1, 60.1) | 59.6 | (55.7, 63.1) | 63.3 | (59.6, 67.7) | 1.2 |
| **Women (n=2,831)** |  |  |  |  |  |  |  |  |  |  |  |
| **Body mass index (kg/m2)** | 20.7 | (17.5, 16.5) | 23.1 | (22.6, 23.6) | 25.1 | (24.6, 25.6) | 27.5 | (26.9, 28.3) | 32.4 | (30.5, 35.3) | 1.6 |
| Weight (kg) | 57.1 | (53.6, 60.1) | 63.4 | (60.3, 66.7) | 68.7 | (65.7, 72.4) | 74.9 | (70.9, 79) | 88.4 | (81.8, 97.9) | 1.5 |
| Waist circumference (cm) | 71 | (68, 75) | 76 | (73, 80) | 82 | (78, 87) | 87 | (83, 92) | 101 | (95, 107) | 1.4 |
| Hip circumference (cm) | 93 | (90, 96) | 98 | (95, 100) | 102 | (98, 105) | 106 | (102, 109) | 114 | (109, 120) | 1.2 |
| Android fat (kg) | 0.8 | (0.6, 1.1) | 1.3 | (1.1, 1.6) | 1.8 | (1.5, 2.1) | 2.3 | (2.0, 2.7) | 3.6 | (3.0, 4.3) | 4.5 |
| Visceral fat (kg) | 0.1 | (0.05, 0.2) | 0.2 | (0.01, 0.3) | 0.3 | (0.2, 0.5) | 0.5 | (0.3, 0.7) | 1.1 | (0.8, 1.5) | 11 |
| Abdominal subcutaneous fat (kg) | 0.7 | (0.5, 0.9) | 1.1 | (0.9, 1.3) | 1.4 | (1.2, 1.7) | 1.7 | (1.5, 2.0) | 2.5 | (2.0, 3.0) | 3.6 |
| Arm fat (kg) | 1.8 | (1.5, 2.1) | 2.3 | (2.0, 2.6) | 2.8 | (2.5, 3.1) | 3.2 | (2.9, 3.6) | 4.2 | (3.7, 4.9) | 2.3 |
| Gynoid fat (kg) | 3.2 | (2.8, 3.6) | 3.9 | (3.4, 4.4) | 4.6 | (4.1, 5.1) | 5.2 | (4.6, 5.8) | 6.5 | (5.7, 7.4) | 2 |
| Leg fat (kg) | 6.4 | (5.5, 7.3) | 7.8 | (6.7, 8.8) | 9.1 | (8.0, 10.2) | 10.4 | (8.9, 11.8) | 13.1 | (11.0, 15.1) | 2 |
| Total fat (kg) | 15.6 | (13.3, 18.1) | 20.0 | (17.6, 22.4) | 24.6 | (22.1, 26.9) | 28.7 | (26.3, 31.6) | 38.5 | (34.5, 45.1) | 2.5 |
| Total lean body mass (kg) | 38.3 | (35.5, 41.5) | 40.4 | (37.4, 43.1) | 40.9 | (38.5, 43.3) | 42.6 | (39.5, 45.2) | 46.1 | (42.3, 49.9) | 1.2 |
